# 2-(2-Iodo­phen­yl)-1,2,3,4-tetra­hydro­isoquinoline-1-carbonitrile

**DOI:** 10.1107/S1600536811015212

**Published:** 2011-05-20

**Authors:** Yanni Ma, Yifang Sun, Feng Zheng, Wenwen Sun, Le Zhou

**Affiliations:** a College of Science, Northwest Agriculture and Forest University, Yangling 712100, People’s Republic of China; b College of Life Science, Northwest Agriculture and Forest University, Yangling 712100, People’s Republic of China

## Abstract

In the title compound, C_16_H_13_IN_2_, the two benzene rings make a dihedral angle of 67.26 (5)°. The six-membered heterocycle of the tetra­hydro­isoquinoline unit adopts a half-chair conformation. In the crystal, adjacent mol­ecules are linked by pairs of weak inter­molecular C—H⋯N hydrogen bonds, forming inversion dimers. An intra­molecular C—H⋯I close contact is also observed.

## Related literature

For the synthesis of the title compound, see: Ishii *et al.* (1985 ▶). For the biological activity of tetra­hydro­isoquinoline derivatives, see: Abe *et al.* (2005 ▶); Kamal *et al.* (2011 ▶); Lane *et al.* (2006 ▶); Liu *et al.* (2009 ▶); Storch *et al.* (2002 ▶); Wright *et al.* (1990 ▶).
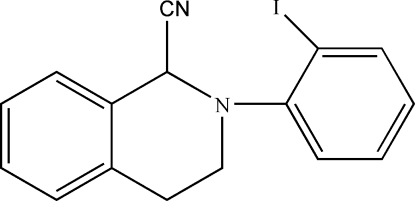


## Experimental

### 

#### Crystal data

C_16_H_13_IN_2_
*M* *_r_* = 360.18
Monoclinic, 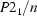

*a* = 11.7607 (12) Å
*b* = 8.4473 (9) Å
*c* = 15.2601 (15) Å
β = 107.662 (1)°
*V* = 1444.6 (3) Å^3^
*Z* = 4
Mo *K*α radiation
μ = 2.20 mm^−1^
*T* = 296 K
0.42 × 0.32 × 0.26 mm

#### Data collection

Bruker APEXII CCD area-detector diffractometer
Absorption correction: multi-scan (*SADABS*; Sheldrick, 1996 ▶) *T*_min_ = 0.458, *T*_max_ = 0.598
10311 measured reflections
2689 independent reflections
2359 reflections with *I* > 2σ(*I*)
*R*_int_ = 0.018

#### Refinement

*R*[*F* ^2^ > 2σ(*F* ^2^)] = 0.035
*wR*(*F* ^2^) = 0.101
*S* = 1.02
2689 reflections
172 parameters
H-atom parameters constrained
Δρ_max_ = 1.16 e Å^−3^
Δρ_min_ = −0.51 e Å^−3^

### 

Data collection: *APEX2* (Bruker, 2004 ▶); cell refinement: *SAINT* (Bruker, 2004 ▶); data reduction: *SAINT*; program(s) used to solve structure: *SHELXS97* (Sheldrick, 2008 ▶); program(s) used to refine structure: *SHELXL97* (Sheldrick, 2008 ▶); molecular graphics: *SHELXTL* (Sheldrick, 2008 ▶); software used to prepare material for publication: *SHELXTL*.

## Supplementary Material

Crystal structure: contains datablocks global, I. DOI: 10.1107/S1600536811015212/is2698sup1.cif

Structure factors: contains datablocks I. DOI: 10.1107/S1600536811015212/is2698Isup2.hkl

Supplementary material file. DOI: 10.1107/S1600536811015212/is2698Isup3.cml

Additional supplementary materials:  crystallographic information; 3D view; checkCIF report

## Figures and Tables

**Table 1:** Hydrogen-bond geometry (Å, °)

*D*—H⋯*A*	*D*—H	H⋯*A*	*D*⋯*A*	*D*—H⋯*A*
C7—H7⋯N2^i^	0.98	2.60	3.418 (5)	141
C7—H7⋯I1	0.98	3.03	3.633 (4)	121

Symmetry code: (i) 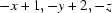.

**Fig. 1.**
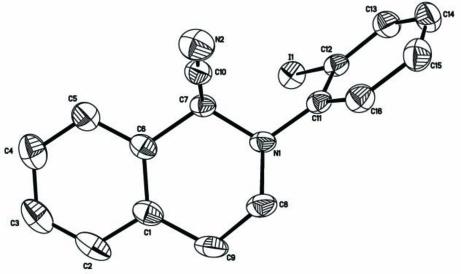
An ORTEP drawing of the title compound, with 30% probability displacement ellipsoids.

**Fig. 2.**
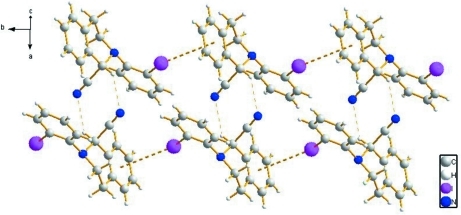
The one-dimension chain structure of the title compound.

## supplementary crystallographic information

### Comment

The tetrahydroisoquinoline derivatives have recently attracted great attention due to their neurotoxicity (Abe *et al.*, 2005; Storch *et al.*, 2002), antitumor activities (Lane *et al.*, 2006; Wright *et al.*, 1990), antimicrobial activity (Kamal *et al.*, 2011; Liu *et al.*, 2009), and so on. With the interests in the synthesis of tetrahydroisoquinoline derivatives with biological activity, we report here the synthesis and crystal structure of the title compound.

As shown in Fig. 1, the molecule of the title compound is built up from one 1-cyan-tetrahydroisoquinoline fragment connected to one 2-iodobenzene ring through the C—N bonds. Benzene C1–C6 and C11–C16 rings are inclined with respect to one another with a dihedral angle of 67.26 (5)°. The conformation of the six-membered ring of tetrahydroisoquinoline fragment is analyzed with respect to the plane formed by C1/C6/C7/C9 and the corresponding deviations of the atoms C8 and N1 are 0.459 (5) and 0.332 (3) Å, respectively. The C—N bonds within the tetrahydroisoquinoline fragment belong to single bond, the inter-ring C—N bond show some π-bond character, while the C—N bond of the cyano is of triple bond character. The important torsion angle which decides the geometry of the title compound is -80.4 (4)° for C12—C11—N1—C7.

In the crystal structure, two adjacent molecules are linked by a weak intermolecular C—H···N hydrogen bond into a dimer. These dimers are further connected by C—I···π interaction into a one-dimension chain along the *b* axis (Fig. 2). I1 aims at the π-cloud of the neighboring benzene ring C1–C6 (*Cg*), The I···*Cg* distance is 3.821 (2) Å with C···*Cg* of 5.622 (5) Å and C—I···*Cg* angle of 141.73°. An intramolecular C—H···I hydrogen bond is also observed (Table 1), which further consolidates the crystal packing.

### Experimental

The title compound was synthesized according to the literature procedure (Ishii *et al.*, 1985), and the single crystals were obtained from a solution of ethyl acetate by slow evaporation at room temperature.

### Refinement

H atoms were treated as riding, with C—H = 0.93–0.98 Å, and with *U*_iso_(H) = 1.2*U*_eq_(C). The highest residual electron density peak is located 1.31 Å from atom C4.

### Crystal data

**Table:**

C_16_H_13_IN_2_	*F*(000) = 704
*M**_r_* = 360.18	*D*_x_ = 1.656 Mg m^−^^3^
Monoclinic, *P*2_1_/*n*	Mo *K*α radiation, λ = 0.71073 Å
Hall symbol: -P 2yn	Cell parameters from 5815 reflections
*a* = 11.7607 (12) Å	θ = 2.6–28.1°
*b* = 8.4473 (9) Å	µ = 2.20 mm^−^^1^
*c* = 15.2601 (15) Å	*T* = 296 K
β = 107.662 (1)°	Block, colourless
*V* = 1444.6 (3) Å^3^	0.42 × 0.32 × 0.26 mm
*Z* = 4	

### Data collection

**Table:**

Bruker APEXII CCD area-detector diffractometer	2689 independent reflections
Radiation source: fine-focus sealed tube	2359 reflections with *I* > 2σ(*I*)
graphite	*R*_int_ = 0.018
φ and ω scans	θ_max_ = 25.5°, θ_min_ = 2.6°
Absorption correction: multi-scan (*SADABS*; Sheldrick, 1996)	*h* = −14→14
*T*_min_ = 0.458, *T*_max_ = 0.598	*k* = −10→10
10311 measured reflections	*l* = −18→18

### Refinement

**Table:**

Refinement on *F*^2^	Primary atom site location: structure-invariant direct methods
Least-squares matrix: full	Secondary atom site location: difference Fourier map
*R*[*F*^2^ > 2σ(*F*^2^)] = 0.035	Hydrogen site location: inferred from neighbouring sites
*wR*(*F*^2^) = 0.101	H-atom parameters constrained
*S* = 1.01	*w* = 1/[σ^2^(*F*_o_^2^) + (0.0558*P*)^2^ + 2.5461*P*] where *P* = (*F*_o_^2^ + 2*F*_c_^2^)/3
2689 reflections	(Δ/σ)_max_ < 0.001
172 parameters	Δρ_max_ = 1.16 e Å^−^^3^
0 restraints	Δρ_min_ = −0.51 e Å^−^^3^

### Special details

**Table:**

Geometry. All e.s.d.'s (except the e.s.d. in the dihedral angle between two l.s. planes)are estimated using the full covariance matrix. The cell e.s.d.'s are takeninto account individually in the estimation of e.s.d.'s in distances, anglesand torsion angles; correlations between e.s.d.'s in cell parameters are onlyused when they are defined by crystal symmetry. An approximate (isotropic)treatment of cell e.s.d.'s is used for estimating e.s.d.'s involving l.s. planes.
Refinement. Refinement of *F*^2^ against ALL reflections. The weighted *R*-factor *wR* and goodness of fit *S* are based on *F*^2^, conventional *R*-factors *R* are based on *F*, with *F* set to zero for negative *F*^2^. The threshold expression of *F*^2^ > σ(*F*^2^) is used only for calculating *R*-factors(gt) *etc*. and is not relevant to the choice of reflections for refinement. *R*-factors based on *F*^2^ are statistically about twice as large as those based on *F*, and *R*- factors based on ALL data will be even larger.

### Fractional atomic coordinates and isotropic or equivalent isotropic displacement parameters (Å^2^)

**Table:**

	*x*	*y*	*z*	*U*_iso_*/*U*_eq_	
C1	0.1617 (4)	1.1100 (5)	0.0837 (3)	0.0552 (10)	
C2	0.0577 (4)	1.1981 (7)	0.0437 (5)	0.0757 (15)	
H2	0.0056	1.2177	0.0778	0.091*	
C3	0.0307 (5)	1.2559 (7)	−0.0439 (5)	0.0821 (17)	
H3	−0.0380	1.3159	−0.0682	0.098*	
C4	0.1052 (5)	1.2253 (7)	−0.0962 (4)	0.0785 (15)	
H4	0.0867	1.2636	−0.1560	0.094*	
C5	0.2082 (4)	1.1369 (6)	−0.0589 (3)	0.0612 (12)	
H5	0.2594	1.1170	−0.0936	0.073*	
C6	0.2349 (4)	1.0783 (5)	0.0296 (3)	0.0470 (9)	
C7	0.3478 (4)	0.9792 (5)	0.0678 (3)	0.0412 (8)	
H7	0.3501	0.9004	0.0213	0.049*	
C8	0.3220 (4)	1.0020 (6)	0.2187 (3)	0.0535 (10)	
H8A	0.3721	1.0956	0.2282	0.064*	
H8B	0.3359	0.9488	0.2774	0.064*	
C9	0.1920 (5)	1.0483 (6)	0.1804 (4)	0.0655 (13)	
H9A	0.1426	0.9568	0.1815	0.079*	
H9B	0.1740	1.1291	0.2194	0.079*	
C10	0.4559 (4)	1.0835 (5)	0.0839 (3)	0.0457 (9)	
C11	0.4559 (3)	0.7998 (4)	0.1851 (3)	0.0417 (8)	
C12	0.4616 (4)	0.6511 (5)	0.1462 (3)	0.0440 (8)	
C13	0.5645 (5)	0.5622 (5)	0.1734 (4)	0.0588 (11)	
H13	0.5677	0.4651	0.1454	0.071*	
C14	0.6628 (4)	0.6160 (6)	0.2419 (3)	0.0622 (12)	
H14	0.7324	0.5559	0.2596	0.075*	
C15	0.6579 (4)	0.7576 (6)	0.2839 (3)	0.0615 (11)	
H15	0.7230	0.7923	0.3317	0.074*	
C16	0.5558 (4)	0.8495 (5)	0.2549 (3)	0.0531 (10)	
H16	0.5539	0.9469	0.2829	0.064*	
I1	0.31094 (3)	0.55223 (3)	0.05008 (2)	0.05981 (15)	
N1	0.3511 (3)	0.8951 (4)	0.1520 (2)	0.0425 (7)	
N2	0.5385 (4)	1.1593 (5)	0.0987 (3)	0.0632 (10)	

### Atomic displacement parameters (Å^2^)

**Table:**

	*U*^11^	*U*^22^	*U*^33^	*U*^12^	*U*^13^	*U*^23^
C1	0.051 (2)	0.047 (2)	0.071 (3)	−0.0028 (19)	0.024 (2)	−0.013 (2)
C2	0.048 (3)	0.070 (3)	0.109 (4)	0.011 (2)	0.025 (3)	−0.021 (3)
C3	0.061 (3)	0.072 (4)	0.099 (4)	0.019 (3)	0.004 (3)	−0.011 (3)
C4	0.075 (3)	0.070 (3)	0.077 (3)	0.013 (3)	0.003 (3)	0.002 (3)
C5	0.060 (3)	0.060 (3)	0.061 (3)	0.005 (2)	0.014 (2)	−0.003 (2)
C6	0.043 (2)	0.038 (2)	0.059 (2)	−0.0019 (16)	0.0148 (18)	−0.0094 (18)
C7	0.0452 (19)	0.0354 (18)	0.047 (2)	−0.0025 (16)	0.0204 (16)	−0.0058 (16)
C8	0.069 (3)	0.049 (2)	0.051 (2)	0.000 (2)	0.031 (2)	−0.010 (2)
C9	0.065 (3)	0.066 (3)	0.082 (3)	0.008 (2)	0.046 (3)	−0.009 (2)
C10	0.046 (2)	0.042 (2)	0.054 (2)	0.0030 (18)	0.0218 (18)	0.0005 (17)
C11	0.051 (2)	0.0360 (19)	0.0430 (19)	−0.0035 (16)	0.0223 (17)	0.0000 (15)
C12	0.053 (2)	0.0389 (19)	0.046 (2)	−0.0035 (17)	0.0227 (17)	−0.0019 (16)
C13	0.073 (3)	0.042 (2)	0.067 (3)	0.008 (2)	0.030 (2)	0.001 (2)
C14	0.058 (3)	0.058 (3)	0.069 (3)	0.014 (2)	0.017 (2)	0.012 (2)
C15	0.057 (3)	0.066 (3)	0.056 (3)	−0.003 (2)	0.010 (2)	0.007 (2)
C16	0.062 (3)	0.048 (2)	0.050 (2)	−0.002 (2)	0.0191 (19)	−0.0051 (19)
I1	0.0717 (2)	0.0453 (2)	0.0607 (2)	−0.01053 (13)	0.01746 (16)	−0.01147 (12)
N1	0.0493 (18)	0.0375 (16)	0.0474 (17)	0.0006 (14)	0.0246 (15)	−0.0060 (14)
N2	0.058 (2)	0.054 (2)	0.082 (3)	−0.010 (2)	0.028 (2)	−0.004 (2)

### Geometric parameters (Å, °)

**Table:**

C1—C6	1.387 (6)	C8—H8A	0.9700
C1—C2	1.403 (7)	C8—H8B	0.9700
C1—C9	1.503 (7)	C9—H9A	0.9700
C2—C3	1.368 (9)	C9—H9B	0.9700
C2—H2	0.9300	C10—N2	1.128 (5)
C3—C4	1.376 (9)	C11—C16	1.390 (6)
C3—H3	0.9300	C11—C12	1.400 (5)
C4—C5	1.390 (7)	C11—N1	1.430 (5)
C4—H4	0.9300	C12—C13	1.377 (6)
C5—C6	1.382 (6)	C12—I1	2.100 (4)
C5—H5	0.9300	C13—C14	1.380 (7)
C6—C7	1.528 (6)	C13—H13	0.9300
C7—N1	1.458 (5)	C14—C15	1.367 (7)
C7—C10	1.505 (6)	C14—H14	0.9300
C7—H7	0.9800	C15—C16	1.386 (7)
C8—N1	1.477 (5)	C15—H15	0.9300
C8—C9	1.513 (7)	C16—H16	0.9300
			
C6—C1—C2	117.4 (5)	H8A—C8—H8B	108.4
C6—C1—C9	120.8 (4)	C1—C9—C8	112.3 (4)
C2—C1—C9	121.7 (4)	C1—C9—H9A	109.1
C3—C2—C1	121.8 (5)	C8—C9—H9A	109.1
C3—C2—H2	119.1	C1—C9—H9B	109.1
C1—C2—H2	119.1	C8—C9—H9B	109.1
C2—C3—C4	120.0 (5)	H9A—C9—H9B	107.9
C2—C3—H3	120.0	N2—C10—C7	177.7 (5)
C4—C3—H3	120.0	C16—C11—C12	117.3 (4)
C3—C4—C5	119.5 (5)	C16—C11—N1	122.6 (3)
C3—C4—H4	120.3	C12—C11—N1	120.2 (3)
C5—C4—H4	120.3	C13—C12—C11	120.8 (4)
C6—C5—C4	120.3 (5)	C13—C12—I1	118.1 (3)
C6—C5—H5	119.9	C11—C12—I1	120.9 (3)
C4—C5—H5	119.9	C12—C13—C14	120.5 (4)
C5—C6—C1	121.0 (4)	C12—C13—H13	119.8
C5—C6—C7	118.8 (4)	C14—C13—H13	119.8
C1—C6—C7	120.2 (4)	C15—C14—C13	119.9 (4)
N1—C7—C10	110.3 (3)	C15—C14—H14	120.1
N1—C7—C6	113.0 (3)	C13—C14—H14	120.1
C10—C7—C6	109.6 (3)	C14—C15—C16	119.8 (4)
N1—C7—H7	107.9	C14—C15—H15	120.1
C10—C7—H7	107.9	C16—C15—H15	120.1
C6—C7—H7	107.9	C15—C16—C11	121.6 (4)
N1—C8—C9	108.0 (4)	C15—C16—H16	119.2
N1—C8—H8A	110.1	C11—C16—H16	119.2
C9—C8—H8A	110.1	C11—N1—C7	112.0 (3)
N1—C8—H8B	110.1	C11—N1—C8	117.1 (3)
C9—C8—H8B	110.1	C7—N1—C8	111.1 (3)
			
C6—C1—C2—C3	−2.3 (8)	C16—C11—C12—I1	173.5 (3)
C9—C1—C2—C3	179.2 (5)	N1—C11—C12—I1	−7.4 (5)
C1—C2—C3—C4	1.4 (9)	C11—C12—C13—C14	2.1 (7)
C2—C3—C4—C5	−0.6 (9)	I1—C12—C13—C14	−174.6 (4)
C3—C4—C5—C6	0.8 (8)	C12—C13—C14—C15	0.7 (7)
C4—C5—C6—C1	−1.8 (7)	C13—C14—C15—C16	−2.5 (7)
C4—C5—C6—C7	179.0 (4)	C14—C15—C16—C11	1.5 (7)
C2—C1—C6—C5	2.5 (7)	C12—C11—C16—C15	1.3 (6)
C9—C1—C6—C5	−179.0 (4)	N1—C11—C16—C15	−177.8 (4)
C2—C1—C6—C7	−178.3 (4)	C16—C11—N1—C7	98.6 (4)
C9—C1—C6—C7	0.2 (6)	C12—C11—N1—C7	−80.4 (4)
C5—C6—C7—N1	−166.6 (4)	C16—C11—N1—C8	−31.4 (5)
C1—C6—C7—N1	14.2 (5)	C12—C11—N1—C8	149.5 (4)
C5—C6—C7—C10	70.0 (5)	C10—C7—N1—C11	−58.6 (4)
C1—C6—C7—C10	−109.2 (4)	C6—C7—N1—C11	178.4 (3)
C6—C1—C9—C8	19.0 (6)	C10—C7—N1—C8	74.5 (4)
C2—C1—C9—C8	−162.6 (4)	C6—C7—N1—C8	−48.5 (4)
N1—C8—C9—C1	−51.5 (5)	C9—C8—N1—C11	−161.5 (4)
C16—C11—C12—C13	−3.1 (6)	C9—C8—N1—C7	68.1 (4)
N1—C11—C12—C13	176.0 (4)		

### Hydrogen-bond geometry (Å, °)

**Table:**

*D*—H···*A*	*D*—H	H···*A*	*D*···*A*	*D*—H···*A*
C7—H7···N2^i^	0.98	2.60	3.418 (5)	141
C7—H7···I1	0.98	3.03	3.633 (4)	121

Symmetry codes: (i) −*x*+1, −*y*+2, −*z*.
